# Flexible Organic Electronic Ion Pump for Flow‐Free Phytohormone Delivery into Vasculature of Intact Plants

**DOI:** 10.1002/advs.202206409

**Published:** 2023-03-19

**Authors:** Iwona Bernacka‐Wojcik, Loïc Talide, Ilaria Abdel Aziz, Jan Simura, Vasileios K. Oikonomou, Stefano Rossi, Mohsen Mohammadi, Abdul Manan Dar, Maria Seitanidou, Magnus Berggren, Daniel T. Simon, Klas Tybrandt, Magnus P. Jonsson, Karin Ljung, Totte Niittylä, Eleni Stavrinidou

**Affiliations:** ^1^ Laboratory of Organic Electronics Department of Science and Technology Linköping University Norrköping SE‐601 74 Sweden; ^2^ Umeå Plant Science Centre Department of Forest Genetics and Plant Physiology Swedish University of Agricultural Sciences Umeå 90183 Sweden; ^3^ Wallenberg Wood Science Center Department of Science and Technology Linköping University Norrköping SE‐60174 Sweden

**Keywords:** bioelectronic devices, drug delivery, polyelectrolytes, photo‐crosslinking, plants vasculature

## Abstract

Plant vasculature transports molecules that play a crucial role in plant signaling including systemic responses and acclimation to diverse environmental conditions. Targeted controlled delivery of molecules to the vascular tissue can be a biomimetic way to induce long distance responses, providing a new tool for the fundamental studies and engineering of stress‐tolerant plants. Here, a flexible organic electronic ion pump, an electrophoretic delivery device, for controlled delivery of phytohormones directly in plant vascular tissue is developed. The c‐OEIP is based on polyimide‐coated glass capillaries that significantly enhance the mechanical robustness of these microscale devices while being minimally disruptive for the plant. The polyelectrolyte channel is based on low‐cost and commercially available precursors that can be photocured with blue light, establishing much cheaper and safer system than the state‐of‐the‐art. To trigger OEIP‐induced plant response, the phytohormone abscisic acid (ABA) in the petiole of intact *Arabidopsis* plants is delivered. ABA is one of the main phytohormones involved in plant stress responses and induces stomata closure under drought conditions to reduce water loss and prevent wilting. The OEIP‐mediated ABA delivery triggered fast and long‐lasting stomata closure far away from the delivery point demonstrating systemic vascular transport of the delivered ABA, verified delivering deuterium‐labeled ABA.

## Graphical abstract

Flexible and robust organic electronic ion pump, an electrophoretic drug delivery device, is developed based on polyimide‐coated glass capillaries. This device is applied for controlled, fluid‐free delivery of stress hormone abscisic acid to the vascular tissue of intact *Arabidopsis* plant, inducing long‐distance closure of stomata. This drug‐delivery device can be used for the fundamental studies and engineering of stress‐tolerant plants.

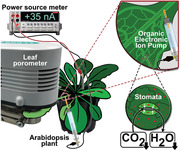

## 1 Introduction

Changing climate and population growth impose the need to better understand how plants respond to environmental stress and ultimately enhance their stress tolerance.^[^ ^1^ ^]^ Plants are sessile organisms that have to acclimate and adapt to their growth environment. They have therefore developed stress‐coping mechanisms in the tissue directly exposed to stress but also in distant tissues in order to prevent further damage. Long distance signaling in plants is mediated via biomolecules such as phytohormones, reactive oxygen species (ROS), calcium waves, and electrical signals.^[^ ^2^, ^3^ ^]^ Plants vasculature creates an integrated microfluidic network that is used by the plant not only for the transport of water and nutrients but also for the transport of signaling molecules from the biosynthetic site to the site of action. Targeted controlled delivery to the vascular tissue can be therefore a biomimetic way to introduce signaling molecules to plants and induce long distance responses. The most common method for delivery in the vascular system is the petiole feeding where a petiole of a detached leaf is immersed in the biomolecules solution, that reaches the leaf apoplast via the transpiration stream.^[^ ^4^, ^5^ ^]^ Due to technical limitations, detached leaves and shoots have been so far widely used in plant biology. However, recently multiple discrepancies have been observed between results obtained using whole plants or plant parts, pointing toward the importance of plant integrity and whole plant communication.^[^ ^6^ ^]^ The petiole feeding method can be adapted for in vivo delivery by connecting a trimmed petiole to a feeding apparatus that contains the solution of interest.^[^ ^7^ ^]^ However, most of the injection/feeding methods require robust plant structures, thus are suitable mainly for larger plant models and are not applicable to the most commonly used plant model, *Arabidopsis thaliana*.^[^ ^8^ ^]^ The needle injection is often performed to the tree vascular system (so‐called “trunk injection”), but this method can cause injuries such as cambial damage, and bark lesion (bark can separate and split). Therefore all these methods are highly invasive, lack dynamic control, induce shear stress, and disturb the internal sap concentration as large amount of water is taken up together with the biomolecules. Recently, a silk‐based photoinjector was shown to deliver various biomolecules to the vascular tissue of intact plants.^[^ ^9^ ^]^ However, this method relies on the dissolution of the silk‐based needles into the vascular tissue and therefore is limited to predetermined released profiles for specific time.

The organic electronic ion pump (OEIP) is a promising technology for electronically controlled delivery of biomolecules without fluid flow.^[^ ^10^ ^]^ The OEIP was initially developed by Berggren and co‐workers^[^ ^11^ ^]^ and has been applied in numerous in vitro biomedical studies^[^ ^12^, ^13^, ^14^ ^]^ and also in vivo interfacing with animal models for controlling neural activity.^[^ ^15^, ^16^ ^]^ Moreover, the OEIP has been applied to plants, for controlled delivery of the phytohormones auxin^[^ ^17^ ^]^ and cytokinin^[^ ^18^ ^]^ at the proximity of the root of *Arabidopsis thaliana*, demonstrating bioelectronic control of root growth. Typically, the OEIP consists of a polyelectrolyte channel that connects source and target electrolytes. Electronic addressing of the device drives ions from the source to the target electrolyte in a controlled manner. The electronic control enables modulation of both frequency and amplitude of the delivered biomolecules that can mimic biological patterns just by programming the current waveform. The high fixed charge of the polyelectrolyte ensures the transport of counter ions only and prevents the backflow of co‐ions enabling dose control. While typical OEIP designs have planar configuration, a glass capillary‐based OEIP (c‐OEIP) was recently developed that has a better interface with biological tissues.^[^ ^19^ ^]^ In this design the polyelectrolyte is directly photocured within the capillary channel, therefore the glass serves both as substrate and encapsulation, significantly reducing the overall size of the device. Previously, we demonstrated that the c‐OEIP with sub‐100 µm diameter can be inserted in the soft leaf tissue of intact tobacco plants with high spatial resolution and minimal wound response.^[^ ^20^ ^]^ By delivering the phytohormone ABA, one of the main hormones involved in plant stress responses, we locally triggered the closure of stomata, i.ethe pores in the leaves, and revealed previously unreported kinetics of ABA‐induced signal propagation. Still, a main limitation of the c‐OEIP for in vivo interface is its mechanical properties, as it is based on glass capillaries of diameter between 60 and 400 µm, thus the device is brittle and can only be inserted in soft tissues with careful manipulation. Polyimide (PI) coating on glass capillaries is commonly used to protect the capillaries from breaking. However due to the high optical absorption of PI in the UV range, photo‐crosslinking via the polyimide coating is challenging,^[^ ^21^, ^22^, ^23^, ^24^, ^25^, ^26^ ^]^ driving the development of several alternative systems using visible^[^ ^21^, ^24^, ^26^ ^]^ and infrared^[^ ^25^ ^]^ range photo‐initiators and rotating light emitting diodes (LEDs). Yet, these photoinitiators are expensive, have low availability, low efficiency, and/or low water solubility.^[^ ^27^, ^28^ ^]^

**Figure 1:**
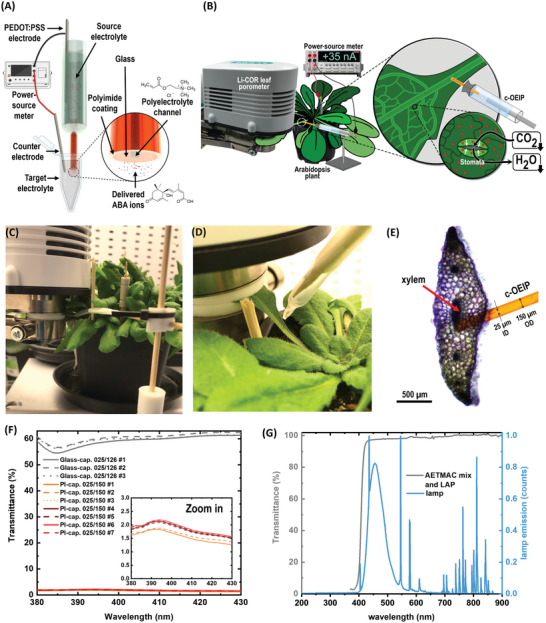
Polyimide‐coated OEIP for advanced petiole feeding assays in intact *Arabidopsis* plants. A) Experimental setup for the OEIP‐mediated ABA delivery in vitro and B) in vivo in the petiole of an intact *Arabidopsis* while monitoring the stomatal conductance with the porometer. C‐D) Photographs of the experimental setup. E) Optical micrograph of the cross‐section of *Arabidopsis* petiole with c‐OEIP inserted indicating the relative proportion of the c‐OEIP in comparison to the targeted petiole and xylem area. F) Transmission spectra of fused‐silica glass capillaries (i.e., “Glass‐cap.”) and polyimide‐coated glass capillaries (i.e., “PI‐cap.”; thickness of polyimide coating: 12 µm per side). The labels refer to the capillaries' inner and total outer diameter (ID/OD). The different lines correspond to the spectra of different capillaries. G) Transmittance spectrum of the polyelectrolyte AETMAC mix solution with the LAP photoinitiator (left *Y*‐axis) and the emission spectrum in normalized counts of the applied blue light (right *Y*‐axis).

In this work, we developed a flexible c‐OEIP that can be inserted in hard tissues and demonstrate in vivo delivery of phytohormones directly in the vasculature of intact plants. The flexible c‐OEIP is based on polyimide‐coated glass capillary which dramatically improves the mechanical properties of the device. The applied polyelectrolyte is based on low‐cost and commercially available precursors, overcoming the need for custom chemical synthesis and greatly broadening the devices’ applicability. To photocure the polyelectrolyte, we used a blue light source and show that enough light penetrates the PI coating reaching the capillary hollow enabling crosslinking of the polyelectrolyte. The device characteristics were determined for ABA, the phytohormone that mediates plant response to drought by triggering the closure of stomata and adaptive physiological/genetic responses (**Figure** **1**A‐E). As a proof of concept, we delivered ABA to the petiole of intact *Arabidopsis* plants inducing a long‐distance response on the stomata and control of transpiration in the targeted leaf.

## 2 Results and Discussion

### 2.1 Development of Polyimide‐Coated c‐OEIPs

To enhance the mechanical properties of the c‐OEIP, we aimed to develop OEIPs based on glass capillaries that are coated with polyimide. PI has outstanding mechanical characteristics; however, it absorbs light significantly.^[^ ^26^ ^]^ Therefore, we first investigated the polyelectrolyte photo‐polymerization in the capillary hollow through the PI coating. Capillaries (25/150 µm ID/OD) were filled with the precursor mixture, consisting of positively charged AETMAC monomer (Figure 1A), PEGDA crosslinker, Igracure 2959 photoinitiator, and the water‐soluble and efficient photoinitiator, LAP.^[^ ^29^ ^]^ For photocuring, we chose a blue light source (400–600 nm) as LAP has absorption tail in blue light region (Figure S1B, Supporting Information) and photopolymerization using visible light is highly attractive due to safety, lack of ozone generation, and higher curing depths.^[^ ^27^, ^30^ ^]^ As the transmittance of the PI‐coated capillaries to light in the blue spectral region is low (Figure 1F and figure S1A, Supporting Information), in order to achieve a high initiation rate, we increased total irradiance by application of a light source of an area significantly larger than the sample and mounted the capillaries in close proximity to the light source (irradiance at 405 nm 3–4 mW cm^−2^). Furthermore, since oxygen inhibits free radical polymerization by reacting with initiator's radicals,^[^ ^31^, ^32^ ^]^ we purged nitrogen into the precursor mix directly before filling the capillary and into the exposure chamber. After 75 min of light exposure periodic bubbles appeared in the capillary channel (Figure S2A, Supporting Information), indicating that the monomers have been polymerized.^[^ ^19^ ^]^ The periodic bubbles were also formed in capillaries with various dimensions of inner and outer diameter, with a longer exposure time needed for capillaries with larger inner diameters (50/150 and 100/200 µm ID/OD, Figure S2B,C, Supporting Information).

**Figure 2:**
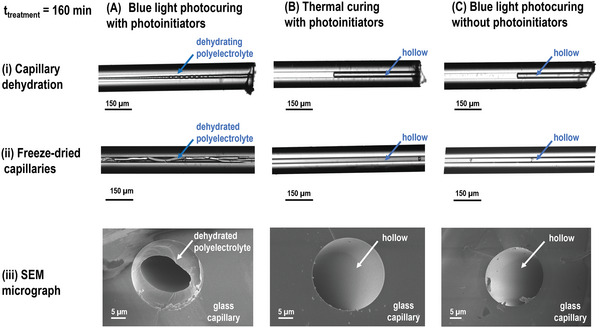
Evaluation of the AETMAC polyelectrolyte crosslinking in the PI‐coated capillaries (25/150 µm ID/OD) after different treatments. The capillaries were treated for 160 min by A) photocuring with photoinitiators; B) thermal treatment in water bath at 75–92 ˚C and C) photocuring without photoinitiators. The formation of the AETMAC hydrogel in the capillary hollow was evaluated by the optical micrographs of (i) the capillary drying; (ii) freeze‐dried samples and (iii) SEM micrographs of freeze‐dried capillaries.

To further confirm the formation of the crosslinked polycation, we monitored its dehydration within the capillary and also characterized freeze‐dried samples. In capillaries that were photocured for 75 and 160 min, we observed the formation of a solid matrix during dehydration, **Figure** **2**Ai and Figure S3Ai, Supporting Information. The presence of the crosslinked polyelectrolyte was also confirmed by scanning electron microscopy (SEM) imaging. The SEM micrographs indicate that the hydrogel adheres well to the surface of the glass capillary and occupies only partially the hollow area (Figure 2iii panel A and S3iii, Supporting Information, panel A) since significant hydrogel shrinkage is caused by freeze‐drying.^[^ ^33^ ^]^ The polyelectrolyte in the hydrated state should fill completely the capillary to prevent the presence of water channels and ensure that the delivery of ions occurs through the charged polyelectrolyte.

However, since the PI absorbs most of the light in the blue light region and the optical transmission spectra of the precursor mixture and the emission spectra of the lamp do not overlap significantly, we proceeded to investigate in more detail the polymerization reaction. As acrylates might undergo thermal self‐polymerization^[^ ^34^ ^]^ (e.g., *n*‐butyl acrylate self‐thermally polymerizes after heating for ˜220 min at 200 ˚C), we first assessed whether the polyelectrolyte crosslinking is caused by temperature increase induced by the light absorption. First, we measured the temperature distribution during the irradiation using an infrared camera. The thermographs show that the temperature of the light bulbs gradually increases during operation reaching a stable temperature of 64 ˚C after ˜15 min of operation (Figure S4, Supporting Information). The temperature increase is observed mainly at the bulb surface while the temperature of the capillary is below 42 ˚C. To verify whether such conditions can induce thermal self‐polymerization of the AETMAC polyelectrolyte, we applied a similar thermal treatment to the capillaries without any blue light illumination. The capillaries were immersed in the water bath heated to 75–92 ˚C, while the capillary ends were placed above the water level. Optical microscopy characterization indicates that after thermal treatment of 160 min, no periodic bubbles were generated in the capillary hollow. During the capillary tip dehydration, instead of the hydrogel shrinking, we observed a homogenous flow of air suggesting that hydrogel was not formed (Figure 2i,ii panel B). SEM analysis confirmed that the capillaries were empty (Figure 2iii panel B), indicating that temperature alone is not sufficient to complete the polyelectrolyte crosslinking in this time window, pointing towards a significant contribution of the light component.

To further explore the polymerization mechanism, we omitted to include the photoinitiators in the polyelectrolyte precursors mix. The lack of periodic bubbles in the channel, capillary drying evaluation, and SEM analysis indicate that just light exposure of the precursors mix without photoinitiator does not induce the AETMAC polymerization (Figure 2C and S3, Supporting Information, panel B).

In summary, AETMAC polycation does not form in the absence of either blue light irradiation or photoinitiators, confirming that in our experimental setup, sufficient blue light penetrates the polyimide coating to complete the polymerization. Although LAP photoinitiator is sensitive mainly in the UV region, its weak optical absorption in the visible light region (see Figure 1G) is sufficient to trigger the polymerization using the emission light that penetrates through the PI coating. Our photocuring system is significantly cheaper and more environmentally friendly than state‐of‐the‐art systems for the polymerization in PI‐coated capillaries that require the application of expensive and nonwater soluble photoinitiators.^[^ ^21^, ^24^, ^25^, ^26^ ^]^

We then investigated the performance of ion loading and delivery of the OEIPs by applying a constant current and measuring voltage changes, since the latter reflects changes in the resistivity of the polyelectrolyte channel. We chose to characterize the devices for the phytohormone ABA as it is one of the main hormones involved in plants stress responses. During loading of ABA anions, the voltage increases as ABA anions are replacing the smaller mobile anions that were compensating for the charges of the AETMAC polycation. When the polyelectrolyte channel is fully loaded with ABA ions, the voltage reaches a plateau. The PI‐coated c‐OEIPs showed very similar loading characteristics to the bare c‐OEIP as it was evaluated for chloride ions (Figure S5, Supporting Information). Since the devices are assembled manually, the capillary length differs between devices, resulting in small differences on the voltage response for the same operating current and ion species. 100% of the devices that were fabricated with 75 min of light exposure were able to satisfactorily load ABA ions (Figure S6, Supporting Information). Interestingly, ≈70% of c‐OEIPs exposed with a higher exposure dose of 120 min became highly resistive during the loading of ABA ions: the voltage gradually increases until reaching the maximum value of the power supply unit (17 V), afterward a decrease of the operating current is observed (Figure S7, Supporting Information). We hypothesize that longer cross‐linking time results in a decrease of the pores’ size hindering the transport of ABA ions. Nevertheless, the hydrated diameter of ABA ions (nanometer range) is much lower than typical SEM resolution, thus further analysis should be carried out to confirm this hypothesis.

**Figure 3:**
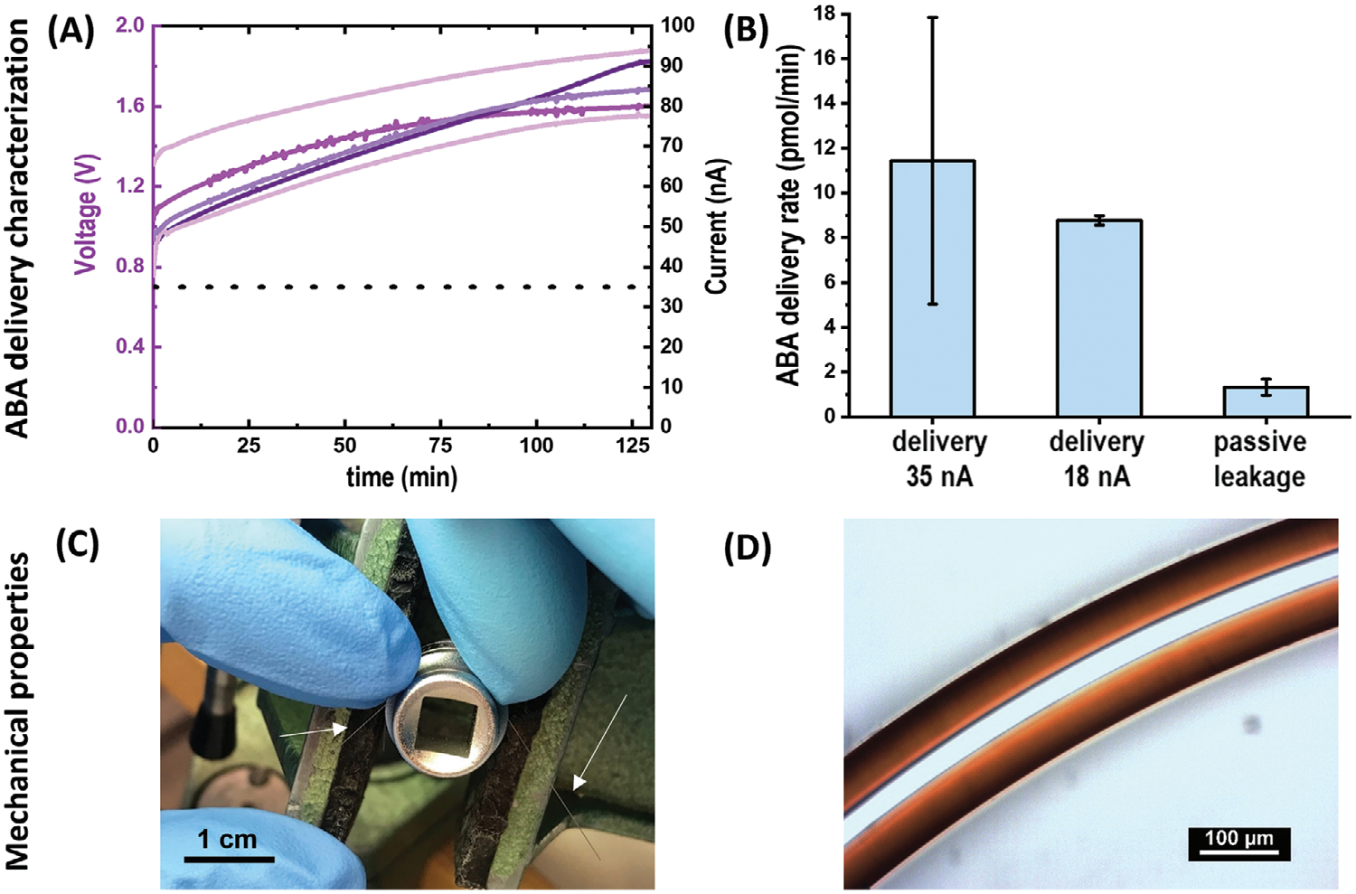
Characterization of ABA delivery and mechanical properties of polyimide‐coated AETMAC c‐OEIP (25/150 µm ID/OD). A) Voltage versus time characteristics during ABA delivery to DI water at constant current of 35 nA. B) In‐vitro ABA delivery rate and passive leakage, as quantified by mass spectrometry (*N* = 5, av ± sd). C) 25/126 µm ID/OD glass capillaries can sustain bending up to a radius of 5.5 mm (the capillary is highlighted by the two white arrows). D) Microscopic image of polyimide‐coated glass capillary (25/150 µm ID/OD) bent to 2.0 mm bending radius (sustainable bending radius: 0.7 mm).

The ABA delivery rate of the developed c‐OEIP was determined in a test solution via mass spectrometry. For operation at a constant current of 35 nA, the delivery rate was 11.4 ± 6.4 pmol min^−1^ giving an electron to ion conversion efficiency of 53% (**Figure** **3**B). Decreasing the operating current by half increased the efficiency to 78% and device to device reproducibility with delivery rate of 8.8 ± 0.2 pmol min^−1^. The ABA delivery efficiency is below 100% due to delivery (co‐transport) of the other anions that are present in the source electrolyte (i.e., hydroxyl ions), as the OEIP delivery is charge‐selective and not ion‐selective.^[^ ^35^ ^]^ The delivery of hydroxyl ions could affect the local tissue pH, however, plant cells have a high buffering capacity.^[^ ^36^ ^]^ Other ionic fluxes that may affect the ABA delivery efficiency is backflow of cations from the target electrolyte (the plant) to the source electrolyte (mainly mobile cations that are present in the petiole at largest concentrations), as the ion‐exchange membrane is not 100% selective. The most abundant cation in the target plant tissue is potassium which is present in the concentration up to 100 mM.^[^ ^37^ ^]^ The small changes induced by the OEIP backflow of the potassium to the source electrolyte are unlikely to significantly affect the intrinsic potassium concentration.

As reported previously, a lower operating current improves the c‐OEIP delivery efficiency,^[^ ^19^, ^38^ ^]^ however to trigger a response in plants, we selected higher operating current as it results in higher ABA doses. The passive leakage rate of the OEIP in the off state was 1.3 ± 0.4 pmol min^−1^ (Figure 3B), resulting in an on/off ratio of 8.8. Overall, the delivery and leakage rates of the AETMAC‐based c‐OEIPs are much lower than in the OEIPs based on hyperbranched polymer.^[^ ^20^ ^]^ We hypothesize that this is related to a smaller pore size or more compact hydrogel network of the linear AETMAC polyelectrolyte in comparison with the one of the hyperbranched polyelectrolyte. Nevertheless, the AETMAC polyelectrolyte is significantly cheaper and, most importantly, is commercially available.

Finally, we evaluated the mechanical properties of the glass capillaries with and without polyimide coating by measuring the sustainable bending radius. The glass capillaries of 150 µm OD and 25 µm ID with polyimide coating could sustain bending down to a radius of ≈ 0.7 mm (Figure S8, Supporting Information). In contrast, the same capillaries without the coating could sustain bending radius down to only 5.5 mm (Figure 3C). Indeed, the glass has a very low fracture toughness and is susceptible to crack formation that initiate from crystal imperfections. In the pristine capillaries, as the micro/nano cracks form, they start to propagate very quickly, undermining the integrity of the whole capillary structure. A microscopic evaluation showed that no cracks were formed in polyimide‐coated capillaries after bending up to a radius of 2.0 mm (Figure 3D). Polymeric coatings improve the mechanical properties of capillaries potentially in several ways, including as a means of filling flaws and for smoothing out the stress distribution around micro/nano cracks on the glass surface during loading.^[^ ^39^ ^]^ It is also believed that the compressive stress on the glass surface caused by shrinkage of the polymer during solidification and curing prevents crack propagation.^[^ ^40^ ^]^

### 2.2 Evaluation of the OEIP Insertion Effect

**Figure 4:**
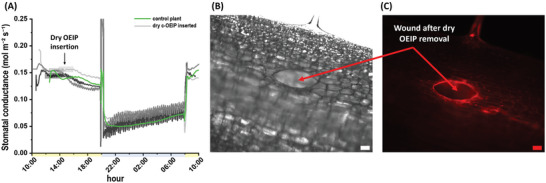
Evaluation of the effect of the dry OEIP insertion into *Arabidopsis* petiole. A) Stomatal conductance response of three wounded plants (grey lines) and typical diurnal stomatal conductance behavior of a representative control plant (green line). The shaded boxes indicate the OEIP insertion time. Different lines correspond to behavior of different plants subjected to the dry‐c‐OEIP insertion. The light and dark periods are marked by yellow and blue fields on time axis, respectively. B) Bright field image representing the wound formed after dry OEIP removal. C) Propidium Iodide staining to detect dead cell in the OEIP‐wounded area. Scale bar: 100 µm.

Any mechanical injury of plant tissue may induce a systemic wounding response. As plants are sessile organisms, when a tissue is locally injured (for example during herbivore attack or leaf detachment) plants have to transmit this information to distant tissues in order for the plant to prepare for the upcoming stress, this is called a systemic response. Plants, however, lack a nervous system therefore long distance signaling in plants occurs in many cases via the vascular tissue with chemical, hydraulic, or even electrical signals.^[^ ^41^ ^]^ As such, development of minimally invasive tools for plant biology is highly needed, while evaluation of their invasiveness is a crucial control experiment. To assess the invasiveness of the OEIP insertion into *Arabidopsis* vasculature we inserted dry devices in the vascular tissue of the leaf petiole. The plant response was evaluated based on the stomatal conductance which is sensitive to vascular tissue disruption. The stomatal conductance was monitored with the LI‐COR leaf porometer (Figure 1B‐D) that measures the water vapor released through the stomata. After OEIP insertion, the plants showed a similar variation of stomatal conductance thought‐out the day as the control plants, i.e., stomatal conductance is higher during day time to enable photosynthesis and lower during nighttime to prevent water loss, **Figure** **4**A. This result indicates that the stomatal conductance is not affected by the dry c‐OEIP insertion and is consistent with our previous work, where c‐OEIP did not cause any substantial response when inserted in tobacco leaf blade.^[^ ^20^ ^]^

We then analyzed the status of the cells surrounding the wound at the site of insertion, via staining with Propidium Iodide, a red fluorescent dye. In plants, the Propidium Iodide stains the cell wall of living cells and the nuclei of dead cells as in the latter case it can pass through the damaged cell membrane.^[^ ^42^ ^]^ Only the perimeter of the insertion site exhibits a bright red fluorescence, indicating the accumulation of the Propidium Iodide in this region, while further away from the wound, no sign of apoptotic processes nor dead cells could be observed (Figure 4B‐C).

Overall, the stomatal conductance response and Propidium Iodide staining show that the insertion of the c‐OEIP in the petiole vasculature does not trigger a systemic response, highlighting the low invasiveness of this approach. This can be attributed to c‐OEIP's small diameter (150 µm OD) that is comparable to the size of a few plant cells; see Figure S9, Supporting Information, and 1E).

### 2.3 c‐OEIP‐Mediated ABA Delivery to Intact Arabidopsis Petiole

**Figure 5:**
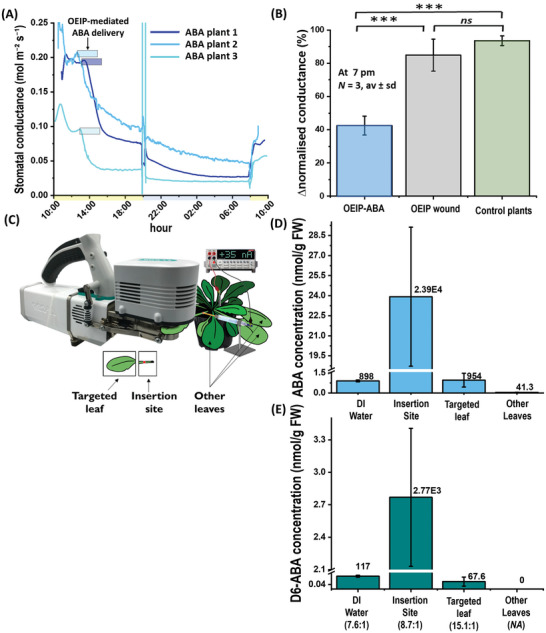
ABA delivered by c‐OEIP to petiole of an intact *Arabidopsis* plants induces stomatal closure far from insertion site. A) Stomatal conductance response to c‐OEIP‐mediated ABA delivery for three different plants (delivery duration is marked by blue boxes). Yellow and blue shade on the time scale indicate light and dark period, respectively. B) Stomatal conductance variations of plants subjected to c‐OEIP‐mediated ABA delivery, plants wounded with dry c‐OEIP, and control plants. Statistical analysis has been carried out by one‐way ANOVA, followed by post‐hoc Tukey test where: ***for *p* < 0.001, **for *p* < 0.005, and *ns* for statistically nonsignificant results. C) The sampling method to investigate in vivo delivery rate and transport of mixture 9:1 of ABA and deuterium labeled ABA (ABA/D6‐ABA). D) Mass spectrometry quantification of c‐OEIP delivered ABA and (E) D6‐ABA ions into a vial of deionized (DI) water, the c‐OEIP insertion site, the targeted leaf, and the other leaves. Below *x*‐axis, the ratio of av_ABA_/av_D6‐ABA_ in analyzed targets is specified.

The OEIP delivers ions locally to the tissue. The ions after their release from the outlet of the OIEP diffuse in the tissue or they can be actively transported via cells. In this study, we targeted the vascular tissue to enable long distance delivery of ions as they enter the plants transpiration stream. To assess the biological applicability of the flexible c‐OEIP, we delivered ABA into the petiole of intact *Arabidopsis* plants and assessed the stomata response with the LI‐COR porometer in real time. We observed that ABA delivery triggered a decrease in stomatal conductance after 1–18 min from the delivery onset (**Figure** **5**A). The stomatal conductance continued to gradually decrease until the end of the day‐time, dropping to a lower value during night‐time, as expected. In the following daytime, the light‐triggered partial stomata opening and the stomatal conductance reached values comparable to the ones at dusk, but significantly lower than the values before the delivery. Therefore, the effect of the c‐OEIP‐mediated ABA delivery lasted for the whole duration of the experiment (≈20 h). The stomatal conductance of ABA‐treated plants was 60% lower and statistically significantly different than the control and wounded plants (Figure 5B). The change in stomatal conductance of the control plants and plants with dry c‐OEIP reflects the natural variation of stomatal conductance during the day. These results clearly show that ABA ions delivered to petiole by c‐OEIP induced the stomata closure in the leaf blade. The effect was very consistent: 3 out of 4 assays showed nearly the same response of stomatal conductance upon ABA delivery. In the fourth assay, the delivered ABA ions still triggered the closure of stomata, but to a reduced extent (Figure S10, Supporting Information). This could be due to the natural variation between plants, to variation in c‐OEIP insertion site, or to ABA delivery rate.

Even though the effect of the OEIP‐mediated ABA delivery on stomatal conductance was evident, we still needed to elucidate whether this was due to the delivered ABA ions that reached stomata via the vascular tissue or whether the stomata closure is triggered by other signaling cascades induced by the delivered ABA ions close to the insertion site. In fact, exogenous ABA application may trigger different processes in plants, such as endogenous ABA biosynthesis,^[^ ^43^ ^]^ generation of reactive oxygen species,^[^ ^44^ ^]^ or modulation of auxin signaling,^[^ ^45^ ^]^ that could contribute to changes in stomatal conductance.

To answer this question, we delivered a solution of standard ABA mixed with deuterium‐labeled ABA (D6‐ABA) that is not endogenously present in plants. The ability of the OEIP to deliver D6‐ABA was evaluated in test solution where a mix of ABA to D6‐ABA was used in the source electrolyte (9:1 by volume). The mass spectrometry analysis of the target solution shows that the c‐OEIP delivers ABA and D6‐ABA with the ratio of 7.6 ± 0.1 to 1 (Figure 5D‐E and Figure S11, Supporting Information). These devices were then inserted in *Arabidopsis* leaf petiole to deliver the ABA/D6‐ABA mix for 30 min triggering fast stomata closure in the leaf blade as it was probed by the porometer. After the delivery, the ABA and D6‐ABA concentrations at the OEIP insertion site, the targeted leaf, and other leaves were analyzed by mass spectrometry (Figure 5C and Figure S10, Supporting Information). Interestingly, in the targeted leaf (leaf blade located from 6 to 40 mm from the OEIP insertion point), D6‐ABA is detected, indicating that the delivered ions were able to travel within 30 min to the leaf blade. At the insertion site, a much higher concentration of ABA and D6‐ABA is observed as the ions diffuse from the c‐OEIP outlet, generating high local concentration. The ratio of ABA to D6‐ABA in the insertion site is 8.7 ± 0.2 to 1, being comparable to the ratio in the source electrolyte. However, there is a significantly higher ratio of ABA to D6‐ABA in the targeted leaf (ratio 15.1 ± 2.3 to 1) that can be due to their different redistribution within the in vivo environment. Finally, in the control leaves, 20 times lower ABA concentration is detected than in the targeted leaf. No D6‐ABA was detected in any of the control leaves indicating that, at least in this time window, the c‐OEIP delivered D6‐ABA did not travel through the whole plant.

Overall, the mass spectrometry results of the ABA/D6‐ABA content along the plant are strong indicators that the observed stomatal closure is due to the delivered ABA ions that travel from the insertion site to the leaf blade via the transpiration stream. As such, the c‐OEIP‐mediated ABA delivery into petiole enabled the control of stomata located up to 40 mm from the c‐OEIP insertion point, while in our previous work in the tobacco apoplast, we could induce stomata closure only up to 5 mm from the c‐OIEP insertion point and with much lower speed. The transpiration stream therefore offers a target for c‐OEIP to induce long‐distance responses.

## 3 Conclusions

In conclusion, we developed a flexible capillary‐based OEIP for controlled delivery of signaling molecules directly in the vasculature of intact plants targeting long distance responses. The c‐OEIP is coated with polyimide that significantly improves the mechanical properties enabling insertion in hard tissues such as the vasculature of plants. The polyelectrolyte channel is based on low‐cost and commercially available precursors that can be photocured with blue light illumination. This protocol can be widely applied without the need for custom chemical synthesis or sophisticated photocuring systems. ABA, a widely studied phytohormone that is involved in many aspects of plant stress responses, can be delivered with the c‐OEIP in a controlled manner and without fluid flow. We delivered ABA to intact *Arabidopsis* petiole triggering fast and long‐lasting stomata closure far away from the insertion site. However, a question that is often overlooked when delivering molecules to plants is whether the observed effect is a direct result of the action of the delivered molecule or is it due to a secondary signaling mechanism. To answer this question, we delivered deuterium‐labeled ABA that was then detected in the leaf area where the stomata closed, far from the c‐OEIP insertion site. This provided direct evidence that c‐OEIP delivered ABA triggered the stomatal closure after being transported in the transpiration stream in the vasculature. Our technology outperforms the current petiole feeding systems where solutions of biomolecule are fed into plants through detached^[^ ^46^ ^]^ or trimmed petioles.^[^ ^7^ ^]^ The c‐OEIP‐based system is far less invasive, and does not disrupt vascular function nor affect the plant integrity. Importantly, with c‐OEIP technology, quantitative, dynamic studies and on‐demand delivery can be achieved due to the electronic control, opening the pathway for advance studies on plant singling that do not rely on genetic engineering. Finally, this technology could be potentially used to prime plants for upcoming predicted stress and enhancing their resistance on demand.

## 4 Experimental Section

### OEIP Fabrication

The 45 cm long sections of polyimide‐coated fused silica capillaries with the outer diameter of 150 µm and inner diameter of 25 µm (TSP025150, Polymicro Technologies LLC, UK) were connected vertically to a nitrogen line via EFD adapter and syringe. The syringe reservoir was connected to a nitrogen supply line to give the flow rate of 0.5 bar. In the first fabrication step, the capillaries were flushed with 2 M KOH for 2 h to allow etching of the inner surface. Next, the capillaries were flushed with DI water for 10 min and dried by N_2_ flushing for 10 min. In the following step, to promote attachment of the monomer to glass surface, vinylic groups were introduced via flushing with *3‐(trimethoxysilyl)propylmethacrylate* (10 wt.% in toluene) for 1 h, followed by ethanol flushing for 20 min and drying through N_2_ flushing for 10 min. The last step was to flush the capillaries with the polyelectrolyte mix containing monomer *2‐(Acryloyloxy)ethyl]trimethylammonium* chloride solution (AETMAC, 35 wt.%, Sigma‐Aldrich), polyethylene glycol diacrylate (PEGDA cross‐linker, molecular weight 575 g mol^−1^, 2 wt.%, Sigma‐Aldrich), the two photoinitiators: *2‐hydroxy‐4’‐(2‐hydroxyethoxy)‐2‐methylpropiophenone* (Igracure 2959; 0.5 wt.%, Sigma‐Aldrich) and Lithium phenyl‐2,4,6‐trimethylbenzoylphosphinate (LAP, 0.5 wt.%, Sigma‐Aldrich) in DI water.

Finally, the monomer in the capillaries was polymerized through photo‐exposure using a homemade box with four blue light lamps DULUX L BLUE 18 (Osram, Sweden). We observed that the crosslinking could be significantly accelerated by decreasing the distance of the capillary to the irradiation source.

Alternatively to the light‐exposure, thermal treatment of the polyelectrolyte was evaluated. The capillary was immersed in a water bath heated to temperature of 75–92 ˚C where the capillary ends were placed at ≈1 cm above water level. The thermal treatment was performed in a cleanroom with yellow lightning (no blue nor UV light emission) under an aluminum foil cover to prevent from the light‐induced reaction.

The c‐OEIP were then stored in NaCl 0.1 M solution, such that they do not dry out, avoiding shrinkage, cracking, and/or void formation within the polyelectrolyte.

### OEIP Assembling

In the device assembling process, the glass capillaries with the photo‐crosslinked polyelectrolyte were cleaved into 10 to 15 mm long samples using a ceramic cleaver. These cleaved capillaries were next attached to polyolefin heat shrink tubing (5–6 cm long), the latter serve as the reservoir for the source electrolyte. The attachment of capillaries was performed by crimping the heated tubing with tweezers after the heat exposure using a heat gun for ≈30 s.

### OEIP Characterization

Optical microscopy images and transmission spectra of the capillaries were recorded using a custom‐built micro‐spectrophotometry setup. The capillary was illuminated using 50 W halogen lamp connected to a microscope (20× objective, Nikon Eclipse L200N, Japan) and the transmitted light was collected from the camera port with an optical fiber (400 µm ID, Hig OH) connected to a spectrometer (Ocean optics Qe Pro, USA). The microscope was equipped with a beam splitter (Thorlabs, SM1CP2) to direct part of the light to a camera and the remaining to the optical fiber, to be able to acquire spectra and images simultaneously.^[^ ^47^ ^]^ We checked that the acquisition area was significantly smaller than the capillary size. An objective lens with 20 × magnification was used to record the spectra. The capillaries were placed on a microscope slide. As a reference, the transmission of the microscope slide was used (the reference was acquired individually for each capillary) and we subtracted the electrical background noise (no light to the detector). To maximize the signal of the spectrometer in the range below 450 nm without saturation at longer wavelengths, the samples were illuminated via 450 nm short‐pass filter (FESH0450, Thorlabs, USA). The light intensity and integration time were adjusted to maximize the counts in the range 390–435 nm, corresponding to the emission tail of the halogen light source (number of counts for the glass reference significantly above the noise level and below photodetector saturation point). As a control, the transmission of glass capillaries stripped from PI were acquired in an identical manner. The polyimide coating of the glass capillaries was stripped by thermal treatment in oven for 2 h at 450 °C.

The transmission spectra of the photoinitiator and polyelectrolyte mix solutions were acquired in the yellow room using optical fiber spectrophotometer Thorlabs CCS200, with a high‐OH optical fiber and a fiber optic cuvette holder and a deuterium light source (SL3, StellarNet). The emission spectrum of the blue light lamp was recorded using the spectrophotometer with the fiber adjacent to the light bulb. The spectra were acquired averaging 20 spectral acquisitions at a time.

The electrical characterization of the OEIPs was carried out with a Keithley 2602 source meter, operated by a customized LabView program, with a two electrodes configuration. Both the electrodes were made of a poly(3,4‐ethylenedioxythiophene):polystyrene sulfonate (PEDOT:PSS) stripes. The loading was carried out by applying a constant current overnight, and the voltage was recorded with the same electrical setup.

### SEM Analysis

The capillaries were cut with ceramic stone into sections of 5–10 mm in length for the SEM analysis to assess the surface morphology of the polyelectrolyte. These sections of each capillary were then frozen in liquid nitrogen and freeze‐dried for 3 days. Each section was then placed vertically in PDMS sample holder with the help of tape to fix their position. All samples were then coated with 20 nm of evaporated gold. A Gemini Sigma 500 Field Emission SEM (Zeiss, Germany) was used for imaging the samples with 3–8 kV of acceleration voltage at 2–4 mm working distance.

### Plant Growth

*Arabidopsis* seeds were hydrated for 48 h at 4 °C and dark condition, then seeded on a mixture of 1:3 vermiculite:soil. Seeds were kept under controlled humidity of 75%–80% and temperature conditions (24 /18 ˚C in the day and night time, respectively), undergoing light/dark cycles of 12/12 h, in a growth chamber. All plants used were 4–6 weeks old.

### OEIP‐ABA Delivery Experiments

Abscisic Acid (ABA) and D6‐ABA (^2^H_6_‐ABA) were purchased from OlChemIm s.r.o. (Czech Republic). ABA and D6‐ABA were dissolved in 5%/95% ethanol/water solution, pH was adjusted up to 5.15 and the solutions were kept frozen until use. ABA solutions were loaded into the target of OEIPs, and a constant current was applied to deliver the ABA ions. A target leaf is inserted into a Li‐COR porometer chamber and the basal stomatal conductance is evaluated. The OEIP was then inserted into the leaf petiole while the stomatal conductance is still recorded, and delivered ABA for 2 h 10 min. Then, the OEIP was removed while the stomatal conductance is still recorded. The whole temporal evolution of the stomatal conductance over time was analyzed with OriginLab software, where the stomata conductance plots were smoothed using adjacent averaging of 20 points.

Stomatal conductance variations (*Δ* normalised conductance) was defined as the difference between normalised stomatal conductance at 12:30 pm (normalised to the stomatal conductance value acquired at 12:30 pm, that was treated as 100%) and 7 pm., i.e., *Δ* normalised conductance = normalised conductance_12:30pm_ – normalised conductance_7pm_ *_._* The statistical significance is carried out using ANOVA tests, with *t*‐student post hoc evaluation.

To determine the position of xylem, the sections of *Arabidopsis* petiole were stained in Toluidine Blue (TBO, Sigma‐Aldrich). The fragile petiole was embedded in 7% agarose to facilitate sectioning. The 100 µm thick cross sections were obtained using a vibratome and stained for 1 min in a water‐based 0.02% TBO staining solution. Additionally, to depict in vivo delivery, OEIP was inserted inside *Arabidopsis* petiole, followed by hand cross sectioning of the area and similar TBO staining, as previously described.

### UHPLC‐ESI‐MS/MS Analysis

Leaf samples were extracted, purified, and analyzed according to method described in Šimura et al.^[^ ^48^ ^]^ Mass spectrometry analysis of D6‐ABA and ABA was performed by an UHPLC‐ESI‐MS/MS system comprising of a 1290 Infinity Binary LC System coupled to a 6490 Triple Quad LC/MS System with Jet Stream and Dual Ion Funnel technologies (Agilent Technologies, Santa Clara, CA, USA). The quantification was carried out in Agilent MassHunter Workstation Software Quantitative (Agilent Technologies, Santa Clara, CA, USA).

### Evaluation of the Effect of OEIP Insertion

*Arabidopsis* leaves underwent the protocol described in the previous OEIP‐ABA delivery section. The leaves were then cut and immersed in a 15 mM solution of Propidium Iodide (Sigma Aldrich) for 10 min. Images were acquired with a Nikon Ti inverted microscope and a standard Texas Red filtering cube. Images were analyzed with ImageJ and OriginLab software.

## Conflict of Interest

M.B., D.T.S., and K.T. are shareholders in the small, researcher‐controlled intellectual property company OBOE IPR AB (oboeipr.com), which owns the patents related to the ion pumps presented above. All other authors declare no conflict of interest.

## Supporting information

Supporting Information

## Data Availability

The data that support the findings of this study are available from the corresponding author upon reasonable request.
