# Supplementary material for: Flexible Organic Electronic Ion Pump for Flow‐Free Phytohormone Delivery into Vasculature of Intact Plants
Source: Adv Sci (Weinh). 2023 Mar 19;10(14):2206409. doi: 10.1002/advs.202206409 (PMC10190655; doi:10.1002/advs.202206409)
Supplement: Supplementary file 1 — Supporting Information [file ADVS-10-2206409-s001.pdf]

ADVANCED SCIENCE
Open Access

Supporting Information

for *Adv. Sci.*, DOI 10.1002/advs.202206409

Flexible Organic Electronic Ion Pump for Flow-Free Phytohormone Delivery into Vasculature of Intact Plants

*Iwona Bernacka-Wojcik, Loïc Talide, Ilaria Abdel Aziz, Jan Simura, Vasileios K. Oikonomou, Stefano Rossi, Mohsen Mohammadi, Abdul Manan Dar, Maria Seitanidou, Magnus Berggren, Daniel T. Simon, Klas Tybrandt, Magnus P. Jonsson, Karin Ljung, Totte Niittylä and Eleni Stavrinidou**

Supplementary Information

Flexible Organic Electronic Ion Pump for flow-free phytohormone delivery into vasculature of intact plants

Iwona Bernacka-Wojcik[1], Loïc Talide[2], Ilaria Abdel Aziz[1], Jan Simura[2], Vasileios K. Oikonomou[1,3], Stefano Rossi[1], Mohsen Mohammadi[1], Abdul Manan Dar[1], Maria Seitanidou[1], Magnus Berggren[1,3], Daniel T. Simon[1], Klas Tybrandt[1,3], Magnus P. Jonsson[1,3], Karin Ljung[2], Totte Niittyla[2] and Eleni Stavrinidou[1,2,3]*

[1] Laboratory of Organic Electronics, Department of Science and Technology, Linköping University, SE-601 74 Norrköping, Sweden

[2] Umea Plant Science Centre, Department of Forest Genetics and Plant Physiology, Swedish

University of Agricultural Sciences, 90183 Umea, Sweden

[3] Wallenberg Wood Science Center, Department of Science and Technology, Linköping University, Norrköping, SE-60174, Sweden

*Correspondence:

eleni.stavrinidou@liu.se

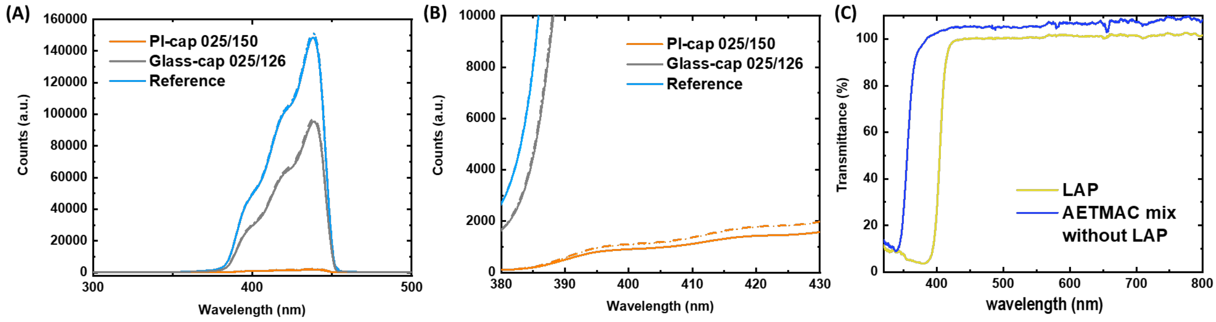

Fig. S1. (A) Number of counts acquired while measuring the transmission spectra of fused-silica glass capillaries (*i.e.* 'Glass-cap') and polyimide-coated glass capillaries (*i.e.* 'PI-cap'; thickness of polyimide coating: 12 µm per side). The labels refer to the capillaries' inner and total outer diameter (ID/OD). The capillaries were mounted on microscope slide, while as the reference, the signal acquired for the microscope slide was used. (B) Zoom in on the range 380-430 nm. The different lines correspond to the spectra of different capillaries or spots on microscope slide for reference. (C) The transmission spectra of the LAP solution and AETMAC mix without LAP acquired in the yellow room using optical fiber spectrophotometer Ocean Optics (model QEPRO-ABS).

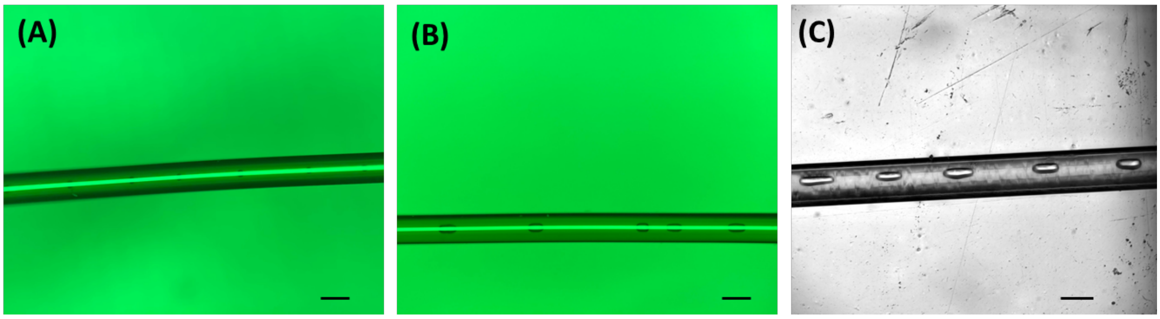

Fig. S2. Optical micrographs of the polyelectrolyte channels crosslinked with the blue light in the polyimide-coated fused silica glass capillaries. The periodic bubbles in the polyelectrolyte have been related to crosslinking of the polyelectrolyte.[1] (A) 25/150 µm ID/OD; (B) 50/150 µm ID/OD; (C) 100/200 µm ID/OD. Scale bar: 150 µm.

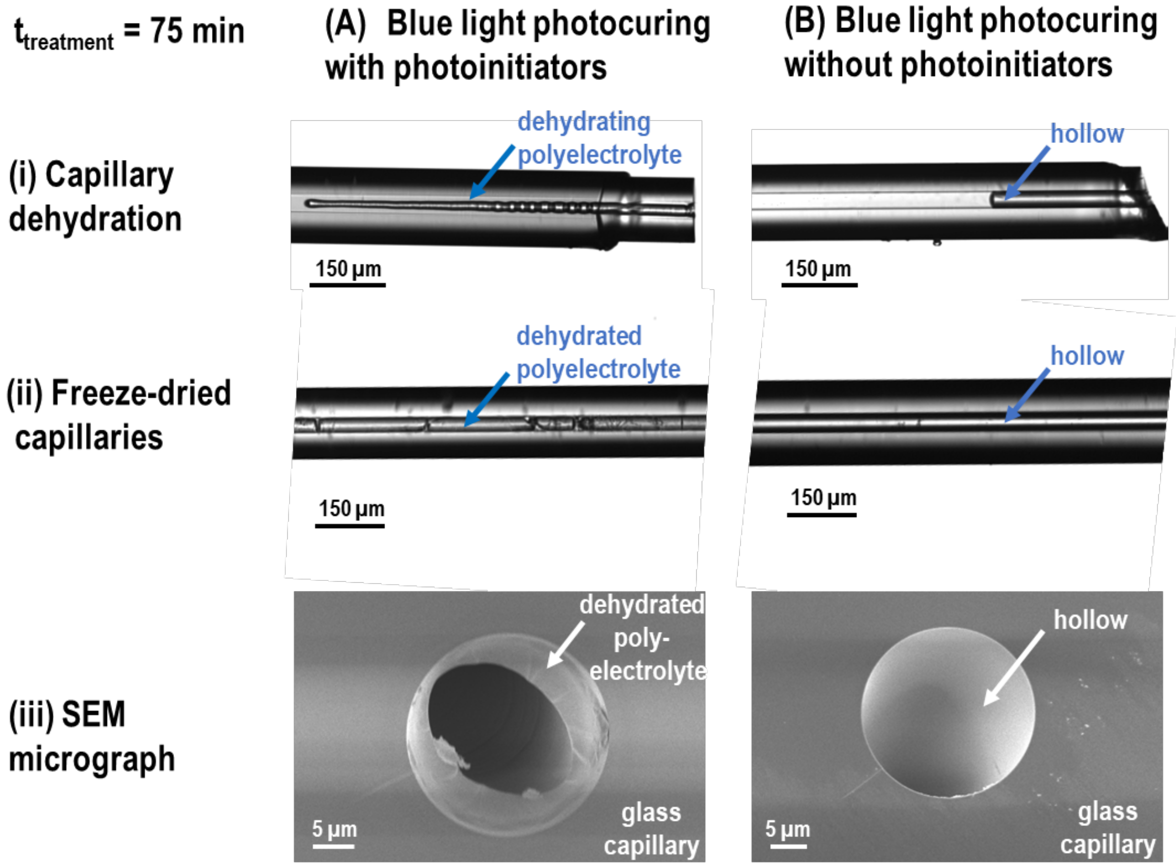

Fig. S3. Characterization of the capillaries (25/150 µm ID/OD) filled with AETMAC polyelectrolyte treated for 75 min by (A) photo-exposure with photoinitiators and (B) photo-exposure without photoinitiators. The formation of the AETMAC hydrogel in the capillary hollow was evaluated basing on the optical micrographs of (i) the capillary drying; (ii) freeze-dried samples and (iii) SEM micrographs of freeze-dried capillaries.

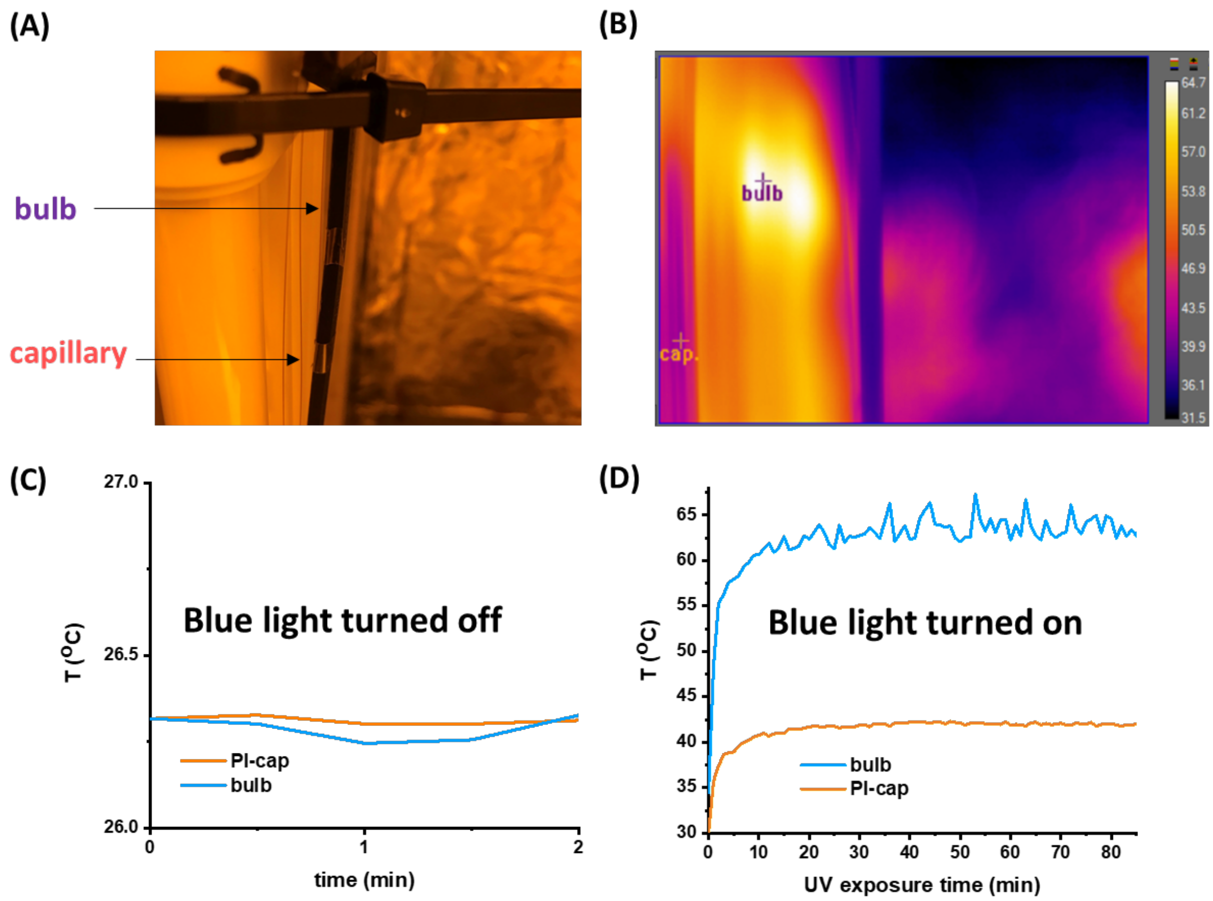

Fig. S4. Infrared camera characterisation of the temperature distribution in the blue chamber during polymerisation of the AETMAC polyelectrolyte in polyimide-coated capillaries. (A) The photography of the blue light chamber interior. (B) Infrared thermography of the chamber interior after 27 min of blue light exposure, indicating the position of the regions of interest. (C) The temporal plot of the temperature of the blue light bulb and capillary with the lights turned off (D) during the exposure.

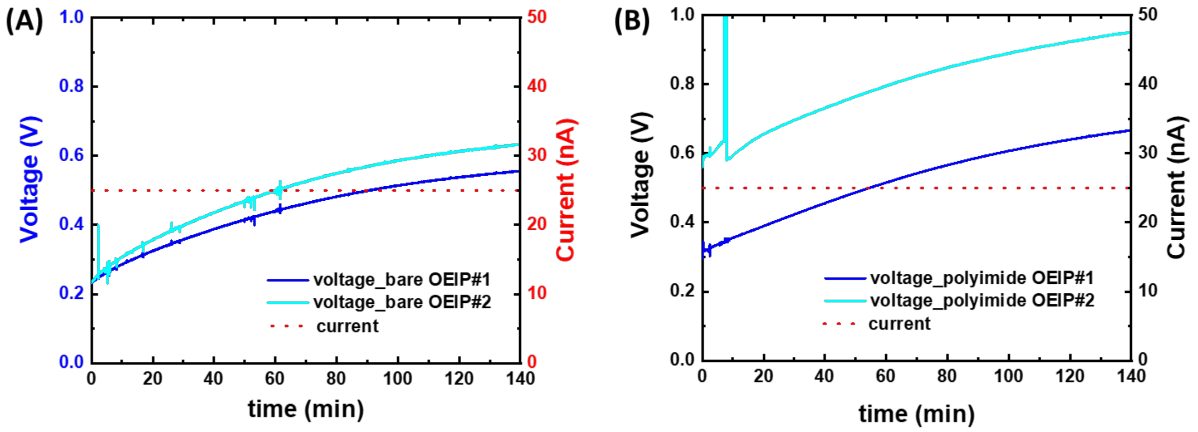

Fig. S5. The current-voltage characteristics of AETMAC OEIPs during loading of chloride ions using glass capillary of 25 µm ID (A) without polyimide coating (126 µm OD) and (B) with polyimide coating (150 µm OD).

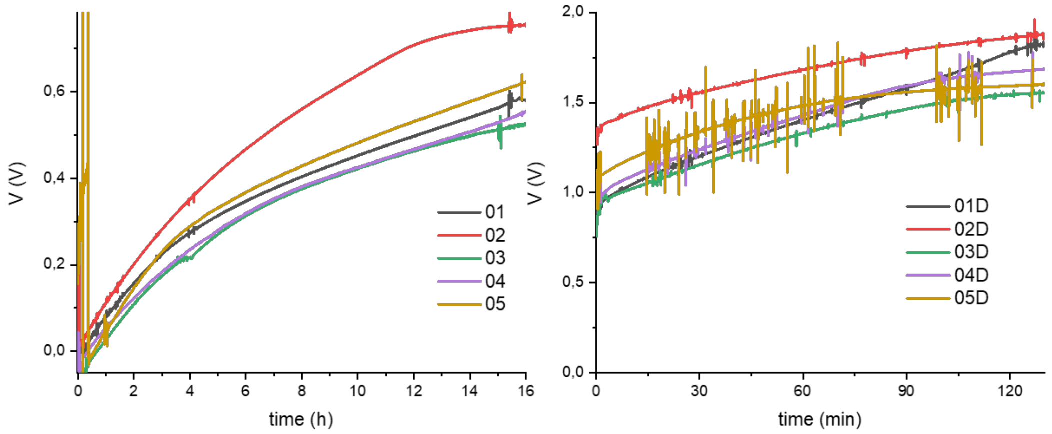

Fig. S6. The performance of the AETMAC c-OEIP of 25 µm ID / 150 µm OD with 75 min of the blue light exposure (100% yield): (left) ABA loading; (right) ABA delivery operated at 35 nA current.

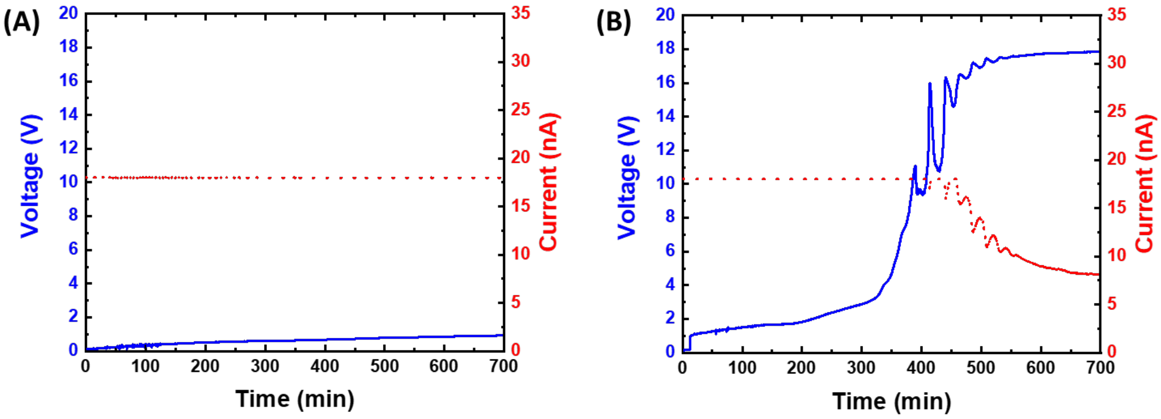

Fig. S7. Current-voltage characteristics of AETMAC c-OEIP devices (150 µm OD / 25 µm ID) operated in the constant current mode when loading ABA ions. (A) The ABA ions are loaded as indicated by constant current behaviour and voltage plateau. 100% of the devices that were fabricated with 75 min of photoexposure were able to satisfactory load ABA ions. (B) Malfunctioning devices: the voltage gradually increases until reaching the maximum value of the power supply unit (17 V), afterwards a decrease of the operating current is observed. ˜70% of c-OEIPs exposed with a exposure dose of 120 min become highly resistive during loading of ABA ions.

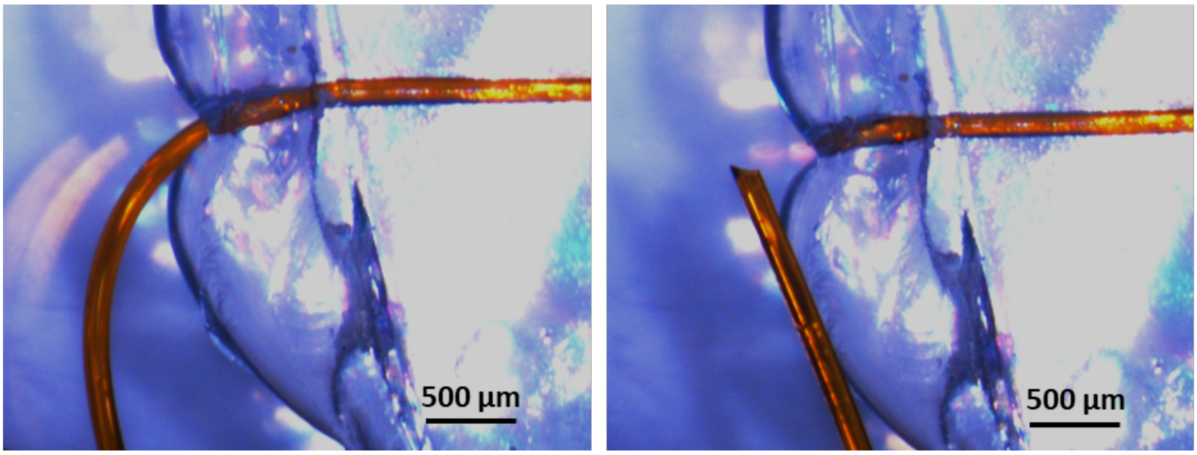

Fig. S8. Microscopic image of polyimide-coated glass capillary (25/150 µm ID/OD) (left) bent to 1.2 mm bending radius (sustainable bending radius: 0.7 mm); (right) Broken capillary.

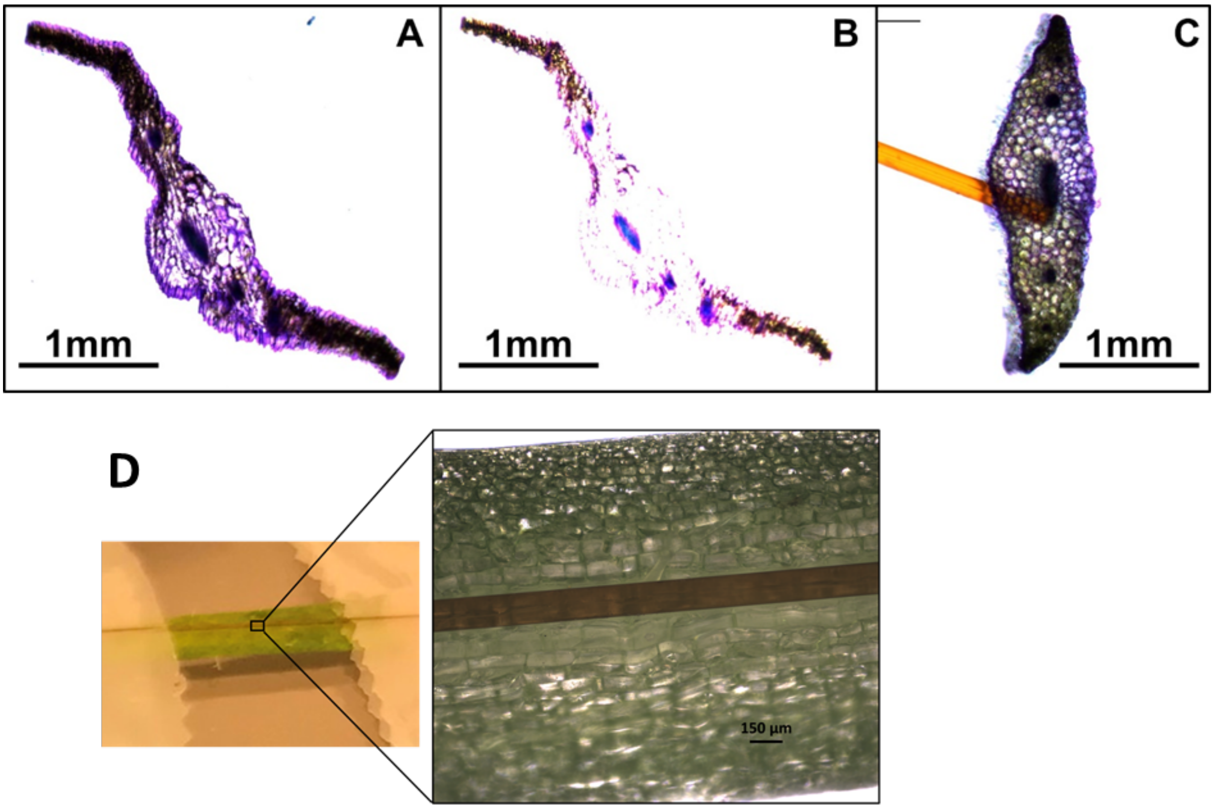

Fig. S9. (A) Microscopic image of toluidine blue-stained section of Arabidopsis petiole. (B) The same section was acquired with higher exposure time to better visualize the position of xylem (stained in blue). (C) The relative proportion of the c-OEIP in comparison to the targeted petiole and xylem area. (D) Photography and optical micrograph of c-OEIP was positioned on the surface of the vein.

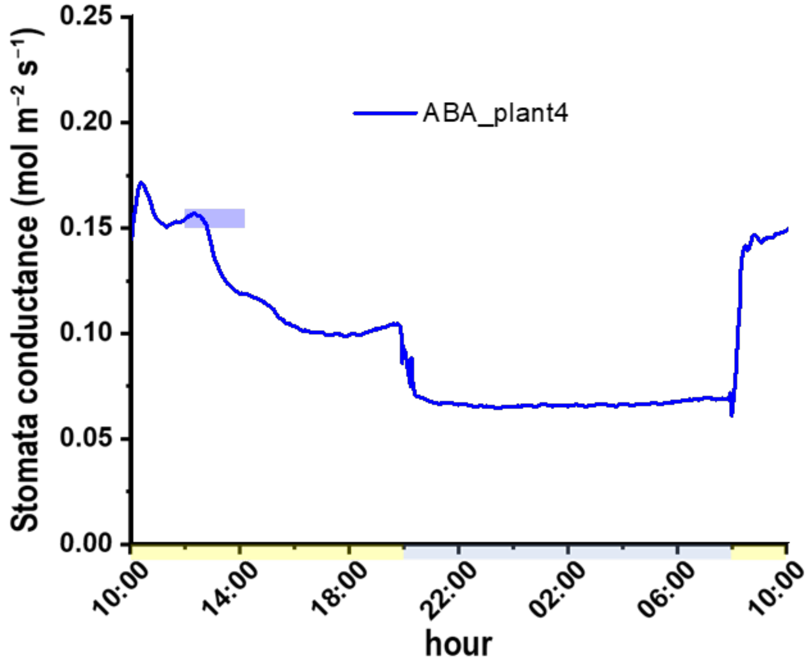

Fig. S10. Stomatal conductance of response to c-OEIP-mediated ABA delivery (2 h 10 min at 35 nA; marked by a blue rectangle). The boxes indicate the c-OEIP insertion time, while the yellow and the blue colors on the time scale indicate the light and dark periods, respectively. The delivered ABA ions triggered the closure of stomata (after 20 min from the delivery onset), but the effect had a different extent. This could be attributed to the natural variation between plants or differences in c-OEIP insertion or the ABA delivery rate.

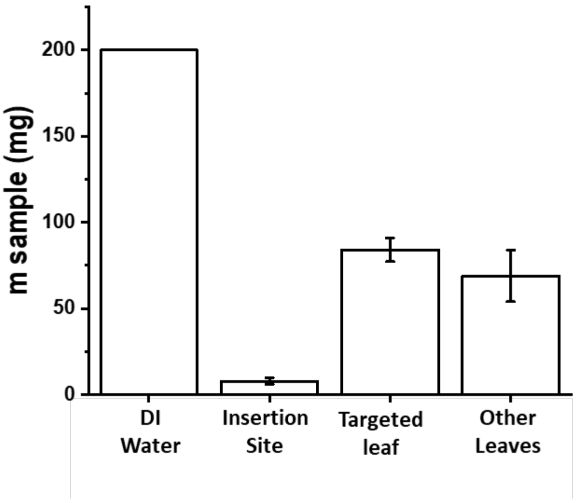

Fig. S11. Mass of the water and leaves (fresh weight) samples used for the analysis of ABA/D6-ABA content (results depicted in Fig. 5C and D).

Bibliography

(1) Poxson, D. J.; Gabrielsson, E. O.; Bonisoli, A.; Linderhed, U.; Abrahamsson, T.; Matthiesen, I.; Tybrandt, K.; Berggren, M.; Simon, D. T. Capillary-Fiber Based Electrophoretic Delivery Device. *ACS Appl. Mater. Interfaces* **2019**, *11* (15), 14200–14207. https://doi.org/10.1021/acsami.8b22680.
